# An Empirical Study of Social Loafing Behavior among Public Officers in South Korea: The Role of Trust in a Supervisor, Perceived Organizational Support, and Perceived Organizational Politics

**DOI:** 10.3390/bs13060498

**Published:** 2023-06-13

**Authors:** Jin Young Kim, Wonho Jeung, Seung-Wan Kang, Ted A. Paterson

**Affiliations:** 1 Suwon City Hall, 241, Hyowon-ro, Paldal-gu, Suwon-si 16490, Republic of Korea; osjiny67@gmail.com; 2 College of Business, Gachon University, 1342 Seongnamdaero, Sujeong-gu, Seongnam-si 13120, Republic of Korea; 3 College of Business, Oregon State University, Austin Hall 326C, 2751 SW Jefferson Way, Corvallis, OR 97331, USA; ted.paterson@oregonstate.edu

**Keywords:** social loafing behaviors, trust in a supervisor, perceived organizational support, perceived organizational politics

## Abstract

This study explored the effects of trust in a supervisor (TIS) on social loafing behaviors of employees. In addition, this study examined the mediating effect of perceived organizational support (POS) on the relationship between trust in a supervisor and employees’ social loafing behaviors. It also examined the moderating effects of perceived organizational politics (POP) on the relationship between TIS and POS, TIS and social loafing behaviors, and POS and social loafing behaviors. Data were collected from local government employees in Korea, and the final sample was 260. Our results indicate that trust in a supervisor has indirect negative effects on social loafing behaviors mediated by POS. In addition, it was found that the effects of TIS on POS and POS on social loafing behaviors were moderated by POP. The results of this study contribute to the extant literature on social loafing behaviors. Moreover, the findings imply that political behaviors in organizations might induce social loafing behaviors.

## 1. Introduction

Modern organizations inhabit fast-changing environments, highlighting the need for organizational members to actively participate in organizational processes and tasks [1]. As a result, much attention has been paid to the positive attitudes and behaviors, such as job satisfaction, organizational commitment, and organizational citizenship behaviors [2]. However, given that much of the organizational work takes place in teams, the study of counterproductive behaviors that undermine team effectiveness is also of high importance [3,4]. One such phenomenon is social loafing, or the loss of motivation to put in efforts towards the achievement of group goals [5,6], which is a widely accepted explanation for group productivity loss [7,8,9,10]. In this study, we aim to explore leadership and organizational mechanisms through which social loafing behaviors can be reduced.

Prior research has explored antecedents of social loafing in groups such as task characteristics [7], organizational justice, and leader–member exchange [11,12], yet much remains to be learned about the leadership and organizational levers that can help reduce the incidence of social loafing in organizations. For example, despite a long-standing body of literature stressing the importance of trust in leaders and managers [13,14,15,16,17], there are few studies which have explored how trust in leadership can potentially reduce social loafing in organizations.

The present study explores two research questions in a public organization in Korea: (1) how does trust in supervisors reduce social loafing behaviors of organizational members and (2) how is the effect of trust in supervisors and perceived organizational support on social loafing behaviors moderated by perceptions of organizational politics? With regards to the first research question, we posit that, consistent with social exchange theory, followers who trust their leaders will be less likely to engage in social loafing behaviors. Furthermore, we explore a mechanism through which trust in a supervisor has its effect on social loafing: perceived organizational support. As previous studies in POS theory have suggested [18,19], we argue that as the most proximal organizational representatives, supervisors influence followers’ perceptions of the overall supportiveness of the organization. Thus, enhanced trust in supervisors is expected to increase followers’ perceptions of organizational support, which, in turn, reduces the social loafing behaviors of organizational members. 

In addition, we investigate the moderating effect of perceived organizational politics on these relationships. Previous studies on the perceptions of organizational politics have confirmed that organizational politics negatively influence important organizational outcomes such as job satisfaction, tension, mood, and performance when organizational members ruminate on organizational politics [20]. While there are studies which have explored the simultaneous effects of politics and support on the attitudes, behaviors and performance of organizational members, there is no previous study [21,22], to our knowledge, which has explored the interaction effects of trust in a supervisor and organizational politics as well as organizational politics and support. Therefore, we introduce perceptions of organizational politics as a critical moderator of the relationship between trust in a supervisor and perceived organizational support, as well as perceived organizational support and social loafing behaviors. 

In creating our research model, we applied and integrated trust theory, the theory of perceived organizational support and social exchange theory. Thus, the results of this study will have theoretical implications in that it will broaden the scope of application of existing theories in explaining social loafing behaviors. 

## 2. Theoretical Background and Hypotheses

### 2.1. Trust in Supervisor and Social Loafing

Social loafing is defined as the tendency of individuals to reduce their individual level of effort when they work in groups [5,23]. The phenomenon was initially discovered in an experiment conducted by Ringelmann [24], wherein he found that the productivity of the group is lower than the aggregation of the productivity of group members. He provided two reasons for the loss of productivity: coordination loss and motivation loss. Coordination loss occurs when there is a misfit between the level and direction of coordinated effort and the moment of maximum effort of individuals within groups. Motivation loss occurs because individuals tend to be dependent on other members and believe that their colleagues will put more efforts into accomplishing the goals of their groups. Latane et al. [6] restated this motivation loss as social loafing. Social loafing is detrimental to organizational performance as well as to cohesiveness, potency and group satisfaction [25]. 

While initial studies, which examined the antecedents of social loafing, were largely conducted in laboratory settings, the growing body of research has examined factors which cause social loafing in actual work groups [7,12,26]. George [7] found that task characteristics such as visibility and intrinsic task involvement are negatively associated with social loafing. Kidwell and Bennett [27] found alternative motives for engaging in social loafing such as rational choice, normative conformity, and affective bonding. Murphy et al. [12] found that interactional justice and leader–member exchange are negatively related to social loafing.

It has also been suggested that supervisors and leaders of groups play an important role in reducing social loafing. For example, Ferrante, Green, and Forster [28] found that groups with formal incentivized leaders outperform those groups without leaders. Building on this and other studies, we posit that trust in a supervisor could be an important factor in reducing social loafing behaviors. Previous studies have examined how trust in a supervisor has positive effects on employees’ attitudes and behaviors, such as turnover intention, organizational commitment, and organizational citizenship behaviors [29,30,31]. Organizational members continuously interact with their colleagues [32,33,34] and, in particular, their interactions with their supervisor have been shown to influence follower attitudes and behaviors [34,35,36,37,38]. Followers build trust in supervisors through their daily interactions with their supervisors [37] and based on the behaviors of supervisors [39]. When trust in their supervisor has been established, followers tend to reciprocate by engaging in positive behaviors such as organizational citizenship behaviors, and showing positive attitudes such as job satisfaction and organizational commitment [39]. Social exchange theory explains these phenomena [40] as followers wanting to repay their leaders’ favor. Accordingly, we argue that followers with high levels of trust in supervisors are less likely to engage in social loafing behaviors.

**Hypothesis** **1.**  *Trust in a supervisor is negatively related to social loafing.*

### 2.2. Mediating Role of Perceived Organizational Support 

Supervisors play significant roles in organizations, including the assignment of tasks to members and the evaluation of their performance [41]. Due to their roles and positions within organizations, supervisors are perceived as agents of the organization rather than as single individuals [18]. The behaviors of supervisors tend to be perceived as those of the organization from the perspective of organizational members [18]. The established trust in supervisors, in turn, is likely to influence followers’ perceptions on how organizations value the contribution of members and provide appropriate rewards for the members. Thus, trust in a supervisor will increase followers’ perceptions of organizational support. Similarly, previous studies have suggested that positive and/or negative behaviors of supervisors are closely related to the perception of organizational support. For example, supervisors’ positive treatment and support results in higher levels of POS [42] whereas abusive supervision reduces POS [42]. 

High levels of POS, in turn, are expected to influence the behaviors of organizational members [43,44]. Positive perceptions of the organization are likely to increase the felt obligation of the members, resulting in positive attitudes and behaviors among employees. Indeed, previous studies have found a positive relationship between POS and organizational citizenship behaviors [45,46,47]. In a similar vein, we argue that enhanced POS is likely to prevent deviant behaviors of organizational members. Thus, we expect that POS reduces the phenomena of social loafing within organizations. In addition, we expect that the effects of trust in supervisors on social loafing will be mediated through heightened POS.

**Hypothesis** **2.**  *Perceived organizational support (POS) is negatively related to social loafing.*

**Hypothesis** **3.**  *Perceived organizational support (POS) mediates the relationship between trust in a supervisor and social loafing.*

### 2.3. Moderating Role of Perceived Organizational Politics

Organizations are inherently political in nature [48], and therefore organizational politics are the reality of organizational life [49,50]. Organizational politics are efforts to enhance one’s personal power or agendas by influencing others through unofficial or informal means. It has been argued that organizational politics are inevitable, since organizational members have different goals, values, and interests and there are almost always limited resources within organizations [48,50,51]. Most organizational members perceive organizational politics as a stressful hindrance, especially in uncertain organizational contexts [52]. Theorists have suggested that organizational politics are perceived to be detrimental because they make the performance–reward relationships ambiguous [21]. In other words, when their colleagues engage in political activities, organizational members feel threatened, because they think their contributions might not be appropriately rewarded [53]. Not surprisingly, then, scholars have found higher levels of conflict and deviance among organizational members in highly political organizational contexts [54]. As a result, previous studies have suggested that people working in highly political organizations tend not to believe that they can accomplish their goals based on their abilities alone [55], making politically-motivated behaviors necessary to cope with and survive in such environments [56]. Consequently, there have been many studies which have explored the negative effects of organizational politics on organizational outcomes such as passive responses [54], threat appraisal [53] and stress-related consequences [57,58,59].

There has also been a series of studies on the relationship between organizational politics and organizational support. Some scholars have suggested that these two constructs are opposite ends of the same construct [60], whereas others have argued that they are related but distinct [20,21]. There have been many studies which explore the simultaneous effects of politics and support on various organizational outcomes [21,58,61]. Cropanzano et al. [21] examined organizational politics and support in relation to work behaviors, attitudes and stress and found that politics is generally related to negative work outcomes while support is related to positive ones. Similarly, Randall et al. [61] found similar results in their study of organizational politics and support predicting job performance and organizational citizenship behavior. Importantly, both Cropanzano et al. [21] and Randall et al. [61] found evidence for the distinctiveness of these two constructs (see also [20]).

Whereas previous studies have found that organizational politics and organizational support are related to work attitudes and organizational citizenship behaviors, they have not examined the interaction effects of organizational politics and organizational support or the interaction effects with other variables. We believe that there could be interaction effects of politics and trust in a supervisor on perceived organizational support and interaction effects with politics and support in predicting and explaining attitudes and behaviors of organizational members. While these interaction effects have been unexplored, Vigoda-Gadot and Talmud [62] found that the effects of organizational politics on job outcomes such as job satisfaction, organizational commitment, stress and burnout were moderated by trust in fellow workers and social support. Similarly, we argue that the effects of trust in a supervisor on perceived organizational support and social loafing behaviors and the effects of perceived organizational support on social loafing would be reduced under highly political contexts. More specifically, the positive effects of trust in a supervisor on perceived organizational support might be decreased when the organizational members are highly political. In a similar vein, the negative effects of trust in a supervisor on social loafing behavior would be reduced when the organizational members are highly political.

**Hypothesis** **4.**  *Perceived organizational politics (POP) moderate the relationship trust in a supervisor(TIS) and perceived organizational support, such that the positive effects of TIS on POS would be stronger for those with low POP.*

**Hypothesis** **5.**  *Perceived organizational politics (POP) moderate the relationship between TIS and social loafing, such that the negative effects of TIS on social loafing would be stronger for those with low POP.*

**Hypothesis** **6.**  *Perceived organizational politics (POP) moderate the relationship between perceived organizational support and social loafing, such that the negative effects of POS on social loafing would be stronger for those with low POP.*

### 2.4. Integrated Model: Moderated Mediation Effect

**Figure 1:**
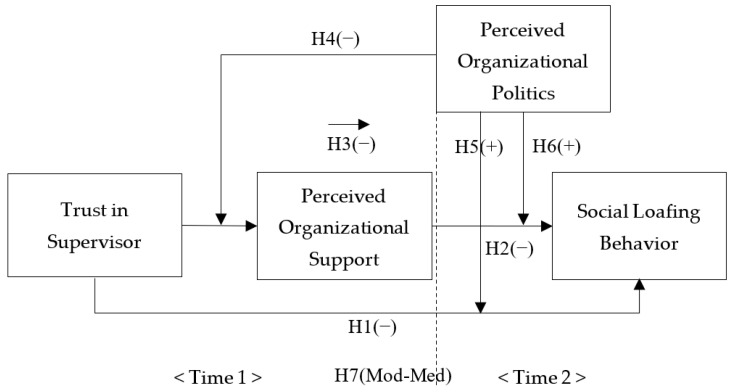
The theoretical research model.

With Hypotheses 3–6 in place, we advance our moderated mediation model of trust in supervisors and social loafing. The effects of trust in a supervisor on social loafing behaviors through perceived organizational support will be reduced under high levels of organizational politics and increased under low levels of organizational politics. Perceived organizational support will be increased by heightened trust in supervisors and decreased by reduced trust in supervisors. Therefore, we expect that trust in a supervisor has increased indirect negative effects on social loafing through perceived organizational support for lower perceptions of organizational politics and decreased indirect negative effects on social loafing through perceived organizational support for higher perceptions of organizational politics. The theoretical research model of this study is depicted in Figure 1.

**Hypothesis** **7.**  *For high perceptions of organizational politics, the indirect effects of trust in supervisors on social loafing through perceived organizational support will be decreased and for low perceptions of organizational politics, the indirect effects of trust in supervisors on social loafing through perceived organizational support will be increased.*

## 3. Methodology

### 3.1. Sample and Procedure

The sample for this study comes from local government officers of a suburban city in South Korea. The participants were asked to complete surveys at two points in time between October and December 2021. The first survey consisted of the measures of trust in a supervisor, perceived organizational support and demographic information. The second survey consisted of the measures of perceived organizational politics and social loafing behaviors. We initially distributed 300 surveys, based on the calculation of the necessary sample size using confidence interval and size of population, and 293 of them were returned during the 1st round of the survey and 275 of them were returned during the 2nd round of the survey. The final sample was 260, after removing incomplete responses (see Appendix A). We think that the high response rate of the survey is attributed to our efforts in encouraging survey participation from the respondents and the cooperative nature of Korean public servants, who willingly participated in the survey. About 55% of the participants were female and a majority of them were college graduates. About 61% of them had worked in the office for more than 10 years, and the average age of the final sample was 37.9 years old.

### 3.2. Measures

The survey participants rated the survey items for the variables on a 5-point Likert scale (scores ranging from 1 = strongly disagree to 5 = strongly agree). Since the original scales were developed in English, we followed the standard back-to-back translation process [63]. The original questionnaires were translated into Korean and reviewed by an expert translator. Then, the Korean-version questionnaires were translated back into English. The validity of the questionnaires was confirmed through the process of back translation. Appendix B contains a comprehensive survey questionnaire.

#### 3.2.1. Trust in a Supervisor

Trust in a supervisor was measured using a 10-item scale adapted from Tan and Tan [64] and Konovsky and Pugh [36]. A sample item is “I feel free to discuss with my immediate supervisor the problems and difficulties I have in my job”. The internal reliability for trust in a supervisor was 0.94. 

#### 3.2.2. Perceived Organizational Support (POS)

Perceived organizational support was measured by adapting a 7-item scale based on the scale developed by Eisenberger et al. [18]. Response options ranged from 1 = strongly disagree to 7 = strongly agree. Sample items include “The organization cares about my well-being”, and “The organization values my contributions to its well-being”. The internal reliability for POS was 0.93.

#### 3.2.3. Perceptions of Organizational Politics (POP)

Perceptions of organizational politics was measured using a 10-item scale developed by Kacmar and Carlson [65]. A sample item is “In this organization, people do what’s best for them, not what’s best for the organization”. The internal reliability for POP was 0.90.

#### 3.2.4. Social Loafing Behavior

Social loafing behaviors were measured using a 7-item scale developed by George [7]. A sample item is “I put forth less effort on the job when other group members are around to do the work”. The internal reliability was 0.83. 

#### 3.2.5. Control Variables

The present study controlled for age, education, rank, and tenure because these variables might influence behaviors of organizational members. These control variables were chosen based on the results of previous studies related to the research variables [7,11,12].

### 3.3. Analytical Method

We analyzed descriptive statistics and correlations using SPSS 19.0 and examined the measurement model using confirmatory factor analysis in order to test the validity of the measurement scales using Mplus 7.0. Finally, we tested hypotheses through the moderated mediation analyses procedures provided by Preacher, Rucker, and Hayes applying latent interaction effects in Mplus 7.0 [66].

## 4. Results

Table 1 shows the descriptive statistics and correlations among the variables used in the study. Social loafing behavior is significantly correlated with trust in a supervisor (r = −0.21, *p* < 0.01), perceived organizational support (r = −0.24, *p* < 0.01) and perceptions of organizational politics (r = 0.25, *p* < 0.01). Trust in a supervisor is positively and significantly correlated with perceived organizational support (r = 0.47, *p* < 0.01) and negatively and significantly correlated with perceptions of organizational politics (r = −0.51, *p* < 0.01). Perceived organizational support was negatively correlated with perceptions of organizational politics (r = −0.49, *p* < 0.01).

### 4.1. Validity and Common Method Bias Checks

We conducted confirmatory factor analysis (CFA) to examine the construct validity of the variables in this study. As shown in Table 2, the hypothesized four factor model has appropriate fit indices compared to the alternative models. The comparative fit index (CFI) was 0.932, and the Tucker–Lewis index (TLI) was 0.926, which exceeded the general cutoff of 0.90 for acceptable fit [67]. In addition, the root-mean-square error of approximation (RMSEA) was 0.050, which was less than the general cutoff of 0.08 for good model fit [67]. As such, all the CFA fit indicators satisfied the general cutoff points. Thus, our hypothesized measurement model was appropriate for further analysis. We also compared the fit indices of our four-factor model with three alternative models. We found that the fit indices of our hypothesized measurement model were significantly better than those of the alternative models. The composite reliability (CR) for all variables met the criteria (CR > 0.7) and all standardized factor loadings on predicted constructs were above the cutoff of 0.50, which indicated the convergent validity of each variable [67,68]. Finally, the correlations among constructs were lower than the square root of AVE, which indicated the discriminant validity of each variable [68].

### 4.2. Hypothesis Testing

Table 3 shows the results of covariance-based SEM with the latent moderating variable. Hypothesis 1 predicts that trust in a supervisor is negatively related to social loafing behaviors. As shown in Table 3, this effect was significant (β = −0.111, *p* < 0.01). Therefore, hypothesis 1 was supported. Hypothesis 2 predicts that perceived organizational support is negatively related to social loafing behaviors of followers. As shown in Model 3, this effect was significant (β = −0.224, *p* < 0.01). Thus, Hypothesis 2 was supported.

Hypothesis 3 predicts that perceived organizational support mediates the relationship between trust in a supervisor and social loafing behaviors. As shown in Table 4, the indirect effect of trust in a supervisor on social loafing behaviors via perceived organizational support was significant (b = −062, BootSE = 0.023, 95%CI = −0.108, −0.016). Therefore, Hypothesis 3 was supported.

Hypothesis 4 predicts that perceptions of organizational politics moderate the relationship between trust in a supervisor and perceived organizational support. As shown in Table 3, the moderating effects of perception of organizational politics was significant (b = −0.182, *p* < 0.05, 95%CI [−0.298, −0.065]), which supports Hypothesis 4.

**Figure 2:**
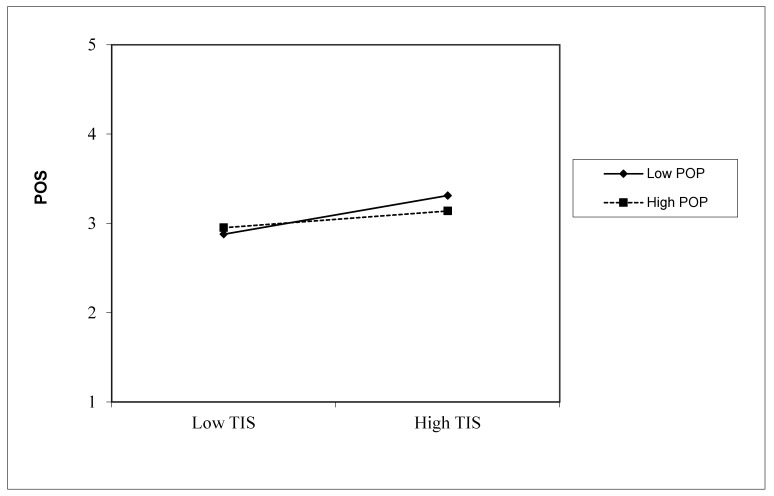
The moderating effect of perceptions of organizational politics on the relationship between trust in a supervisor and perceived organizational support. Note: TIS = trust in supervisor, POP = perceptions of organizational politics.

Figure 2 shows the interaction effects of perceptions of organizational politics and trust in a supervisor on perceived organizational support. The figure shows that the effect of trust in a supervisor on perceived organizational support was only significant when perceptions of organizational politics were low. The simple slope test also indicates that the effects of trust in a supervisor was significant (b = 0.515, *p* < 0.01, 95%CI [0.301, 0.728]) with low-level perceptions of organizational politics, but non-significant (b = 0.151, *p* > 0.05, 95%CI [−0.026, 0.328]) with a high level of perceptions of organizational politics.

Hypothesis 5 predicts that perceptions of organizational politics moderate the relationships between trust in a supervisor and social loafing behavior. As shown in Table 3, the moderating effects of perception of organizational politics was not significant (b = −0.053, *p* > 0.05, 95%CI [−0.173, 0.067]), which does not support Hypothesis 5.

Hypothesis 6 predicts that perceptions of organizational politics moderate the relationships between perceived organizational support and social loafing behaviors. As shown in Table 3, the moderating effects of perception of organizational politics was significant (b = 0.245, *p* < 0.05, 95%CI [0.081, 0.409]), which supports Hypothesis 6.

**Figure 3:**
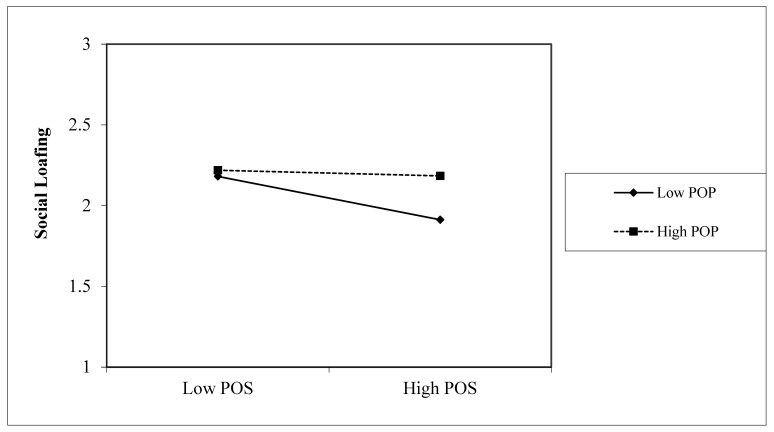
The moderating effect of perceptions of organizational politics on the relationship between perceived organizational support and social loafing. Note: POS = perceived organizational support, POP = perceptions of organizational politics.

Figure 3 shows the interaction effects of perceptions of organizational politics and perceived organizational support. The figure shows that the effect of perceived organizational support was only significant when perceptions of organizational politics were low. The simple slope test also indicates that the effect of POS was significant (b = −0.548, *p* < 0.01, 95%CI [−0.791, −0.305]) with low-level perceptions of organizational politics but non-significant (b = −0.058, *p* > 0.05, 95%CI [−0.263, 0.146]) with high-level perceptions of organizational politics.

Finally, Hypothesis 7 predicts the conditional indirect effects of trust in a supervisor on social loafing behaviors through perceived organizational support. As shown in Table 5, the conditional indirect effects of trust in a supervisor on social loafing behaviors were statistically significant for the low level of organizational politics (b = −0.282, 95%CI: −0.446, −0.118) and medium level of organizational politics (b = −0.101, 95%CI: −0.169, −0.033), but not significant for the high level of organizational politics ((b = −0.009, 95%CI: −0.042, 0.024). Therefore, Hypothesis 7 was also supported.

In this study, we utilized a two-wave time-lagged survey to minimize the potential issue of common method bias. However, the potential issue of common method bias may still exist, since all variables were rated by the same respondents. Therefore, we conducted a single common-method factor approach suggested by Podsakoff et al. [69]. These results indicate that the data of this study likely do not have a serious problem of common method bias [69,70].

## 5. Discussion

### 5.1. Theoretical Contributions

The present study aimed to explore two organizational phenomena: (1) how trust in supervisors reduces social loafing behaviors of organizational members, and (2) how the effect of trust in a supervisor and perceived organizational support on social loafing behaviors is moderated by perceptions of organizational politics. It was found that trust in a supervisor is negatively related to social loafing behaviors of organizational members, which indicates that subordinates tend not to be engaged in social loafing behaviors when they trust their supervisors. It was also found that the effects of trust in a supervisor on social loafing behaviors were mediated by perceived organizational support. This finding is consistent with previous studies which suggested that supervisors tend to be perceived as agents of organizations by the organizational members. Furthermore, this study found that the moderating effects of perceptions of organizational politics were statistically significant for the relationship between trust in a supervisor and perceived organizational support, as well as between the perceived organizational support and social loafing behaviors of organizational members. While organizational members are more likely to reduce social loafing behaviors, this happens only when they perceive that other organizational members do not engage in politically-motivated actions.

The present study has several theoretical and practical implications. First of all, this study contributes to the extant literature on social loafing behaviors by introducing a leadership mechanism (trust) through which social loafing behaviors can be reduced. Previous studies had shown that leader–member exchange relationships played a role in reducing the incidence of social loafing [12], but other studies exploring the role of supervisors and leaders in social loafing behaviors are few and far between. The current study’s finding regarding the link between trust in a supervisor and social loafing is interesting, because at first glance the level of trust between team members would seem to be more important than the level of trust between the team members and the leader in predicting social loafing. However, our findings highlight the important role that supervisors, as the most proximal organizational agents, play in shaping the experience of the members and how they interact with one another towards the achievement of organizational goals. Future studies can compare the impact of trust within the team and its members with our findings here regarding trust between leaders and followers, through the use of social network analysis. While there have been studies on trust in a supervisor in international settings [29,30,31], there have been few studies which have explored the effect of trust in a supervisor on employees’ social loafing behavior.

Second, this study expands the literature dealing with perceived organizational support by establishing its mediating role in the supervisory trust–social loafing relationship. It is meaningful, since this influential process integrates trust theory and POS theory in explaining social loafing behaviors of organizational members. The findings of this study are similar to those of Eisenberger et al. [19], since both withdrawal behaviors and social loafing are detrimental counterproductive behaviors of organizational members. However, in addition to similar findings to their study, we found that the effects of POS are conditional on the level of perceptions of organizational politics, which is another important organizational characteristic.

Third, this study contributes to the literature on perceptions of organizational politics. There has been a long debate about the relationship between perceived organizational support and perceptions of organizational politics [21,54]. While there have been many different opinions about the relationships, we believe that these two constructs are related but, at the same time, distinct [20]. The findings of this study not only support the distinctiveness of those two constructs, but also provide evidence that there are interaction effects of perceived organizational support and perceptions of organizational politics in explaining organizational members’ behaviors. This interaction effect has not been shown in prior research related to these two constructs, and the findings presented here should spur additional research to uncover other situations where these two related constructs interact. In addition, it was also found that the negative effects of POP decrease the positive effect of TIS, which indicates that POP is a significant factor that acts as a boundary condition in organizations.

In summary, this study aimed to integrate trust theory, POS theory, and social exchange theory in explaining social loafing behaviors of organizational members in government organizations in Korea. The theoretical arguments and empirical findings of this study contribute to the expansion of several theories. In previous studies, these theories have been applied to explaining and predicting many organizational outcomes. The findings of this study reinforce the utility of the theories by integrating these theories to explain the phenomena of social loafing in a public organization.

### 5.2. Managerial Implications

This study also provides several practical implications. First of all, this study helps managers to understand how to prevent their subordinates from engaging in social loafing behaviors. The findings of this study suggest that managers should strive to build trusting relationships with their followers, thus adding to the list of benefits that emerge from supervisor–subordinate relationships characterized by trust.

In addition, perceived organizational support would also prevent social loafing phenomena in groups. The findings of our study suggest that managers would be able to increase perceive organizational support by building trust relationships with their followers. Furthermore, managers can increase followers’ perceptions of organizational support by providing reward and training programs for their members.

Third, the findings of this study also suggest that organizational politics hinder the effects of perceived organizational support on social loafing. Therefore, managers need to make organizations less political by increasing transparency in the operational procedures. In this way, managers may be able to reduce the social loafing behaviors of their subordinates.

### 5.3. Limitations and Future Research Directions

While the findings of this study have significant implications, this study also has some limitations that future studies should address. First, while this study measures the independent variable and dependent variable at different points in time, it might be preferable to measure the dependent variable from a different source, such as colleagues or supervisors. In addition, we collected TIS and POS data at the same time from the same source, and there might be a common method bias between these two variables. We conducted a single common-method factor approach suggested by Padsakoff et al. [69] and a serial confirmatory factor analysis, and found that there is likely not a problem with common method bias. Nonetheless, future research can utilize this design and compare and contrast results with the current study. Another useful method to test common method bias is to utilize a marker variable. We did not analyze common method bias using a marker variable, since we do not have an appropriate marker variable. It is also recommended that common method bias should be tested in future studies. There might also be a potential problem of endogeneity or a reverse causal relationship between TIS and POS, since we collected TIS and POS data at the same time. We argued that TIS is an antecedent of POS, based on previous studies such as Eisenberger et al. [18]. Nonetheless, future studies might need to consider alternative research designs to eliminate the potential problem of endogeneity.

Second, we examined the moderating effect of perceptions of organizational politics on the effects of trust in a supervisor and perceived organizational support on social loafing. What we did not include in our model were individual characteristics. Future studies could benefit from exploring how individual differences are related to and interact with organizational characteristics such as organizational politics and support, in explaining the behaviors of organizational members.

Third, this study introduced the interaction effects of perceptions of organizational politics and perceived organizational support. In this study, we treated these variables as individual-level variables, since we collected the data from one organization. However, these constructs can be treated as organizational level constructs, and multi-level analysis can be utilized with samples from multiple organizations.

Fourth, this research study centers on the examination of attitudes and perceptions among Korean public servants, and it is important to consider the potential influence of their cultural heritage on their attitudes and perceptions. Consequently, a cautious interpretation of these findings is necessary, particularly when attempting to extrapolate them to other countries, cultures, and settings. Furthermore, conducting future research that explores the impact of supervisor trust on perceived organizational support and social loafing behavior in culturally heterogeneous nations has the potential to yield novel perspectives and enable the formulation of broader conclusions within different cultural contexts.

## 6. Conclusions

The present study enhances our understanding of the effects of trust in a supervisor and perceived organizational support on the social loafing behaviors of organizational members. The findings in this study confirm that individuals who establish trusting relationships with their supervisors tend not to engage in social loafing behaviors because of an enhanced perception of organizational support. In addition, this study found that perception of organizational politics moderates the effects of perceived organizational support on social loafing. This finding suggests that organizational members have multiple perceptions of their organizations, and these multiple perceptions can simultaneously influence the behaviors of organizational members.

## Figures and Tables

**Table 1:** Means, Standard Deviations, Correlations, and Reliabilities.

	1	2	3	4	5	6	7	8	9
1. Sex									
2. Age	−0.05								
3. Education	0.06	−0.24 **							
4. Rank	−0.03	0.67 **	−0.03						
5. Tenure	0.01	0.81 **	−0.33 **	0.75 **					
6. Trust in Supervisor	−0.07	0.02	−0.04	−0.03	−0.03	(0.944)			
7. POS	0.03	0.03	0.01	0.09	0.01	0.47 **	(0.931)		
8. POP	0.14 *	0.05	0.03	0.08	0.11 †	−0.51 **	−0.49 **	(0.896)	
9. Social Loafing	0.03	−0.05	0.09	−0.04	−0.04	−0.21 **	−0.24 **	0.25 **	(0.851)
Mean	0.55	37.90	2.77	1.93	17.65	3.60	3.07	3.00	2.10
SD	0.50	7.40	0.74	0.72	8.15	0.57	0.59	0.60	0.47

*n* = 260, † < 0.10, * *p* < 0.05, ** *p* < 0.01; Internal reliability coefficients (Cronbach’s alpha) are listed along the diagonal; Sex: 0 = male, 1 = female; Education: 1 = high school diploma, 2 = junior college, 3 = university, 4 = graduate school; Rank: 1 = junior staff, 2 = assistant, 3 = senior staff.

**Table 2:** Results of Confirmatory Factor Analyses.

	χ^2^ (df)	△χ^2^ (△df)	CFI	TLI	SRMR	RMSEA
Research Model (4 factors)	852.390 (518)	-	0.932	0.926	0.056	0.050
Alternative model 1 (3 factors)	1420.836 (521)	568.446 (3) ***	0.816	0.802	0.100	0.082
Alternative model 2 (2 factors)	2001.722 (523)	1149.332 (5) ***	0.698	0.676	0.125	0.104
Alternative model 3 (1 factor)	2600.458 (524)	1748.068 (6) ***	0.576	0.546	0.141	0.123

Notes: *n* = 260, *** *p* < 0.001; CFI = comparative fit index; TLI = Tucker–Lewis index; RMSEA = root-mean-square; 3 factor: TIS + POS, POP, SLB; 2 factor: TIS + POS + POP, SLB; 1 factor: TIS + POS + POP + SLB; TIS = Trust in Supervisor; POS = Perceived Organizational Support, POP = Perceptions of Organizational Politics; SLB = Social Loafing Behavior; Chi-square difference for each model reflects its deviation from the four-factor model.

**Table 3:** Results of Structural Equation Modeling with Latent Interaction Effects.

Path	Estimate	S.E	T	*p*	Lower	Upper
TIS → SLB	−0.111	0.044	−3.047	0.002	−0.219	−0.047
TIS → POS	0.333	0.080	4.142	0.000	0.175	0.491
POS → SLB	−0.303	0.078	−3.876	0.000	−0.457	−0.150
TIS*POP → POS	−0.182	0.059	−3.057	0.002	−0.298	−0.065
TIS*POP → SLB	−0.053	0.061	−0.872	0.383	−0.173	0.067
POS*POP → SLB	0.245	0.084	2.925	0.003	0.081	0.409

Notes: *n* = 260, TIS = trust in supervisor, POS = perceived organizational support, POP = perceptions of organizational politics, SLB = social loafing behavior; AIC = 15,050.958, BIC = 15,453.315.

**Table 4:** Bootstrapping Mediation Results.

	Dependent Variable: SLB			
Mediator	Indirect Effect	SE	95%CI	
			LL	UL
POS	−0.062	0.023	−0.108	−0.016

Note. *n* = 260, Bootstrap sample size = 10,000, POS = Perceived Organizational Support, SLB = Social Loafing Behavior, SE = standard error, CI = confidence interval, LL = lower limit, UL = upper limit.

**Table 5:** Moderated Mediation Results.

Value of POP	Indirect Effect	*SE*	*p*	95%CI	
				LL	UL
−1 *SD*	−0.282	0.084	0.001	−0.446	−0.118
*M*	−0.101	0.035	0.004	−0.169	−0.033
1 *SD*	−0.009	0.017	0.597	−0.042	0.024

Note. *n* = 260, Bootstrap sample size = 10,000, TIS = Trust in Supervisor, POS = Perceived Organizational Support, POP = Perceived Organizational Politics, SLB = Social Loafing Behavior, SE = standard error, CI = confidence interval, LL = lower limit, UL = upper limit.

**Figure A1:**
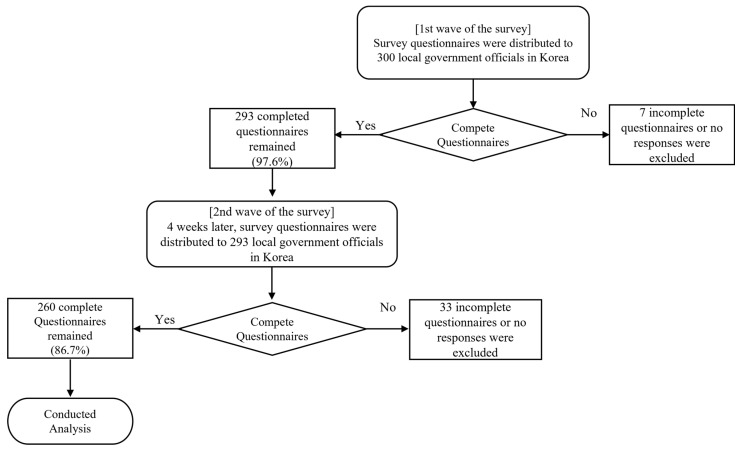
Sampling Procedures.

## Data Availability

The data presented in this study are available upon reasonable request from the corresponding author.

## Appendix B. Measurements

Trust in Supervisor (Cronbach’s alpha = 0.944) [33,61].

1. I am sure that I fully trust my supervisor (0.741).
2. My supervisor is open and upfront with me (0.708).
3. I believe my supervisor has high integrity (0.756).
4. In general, I believe my supervisor’s motives and intentions are good (0.723).
5. My supervisor is always honest and truthful (0.814)
6. I think my supervisor treats me fairly (0.770).
7. I can expect my supervisor to treat me in a consistent and predictable fashion (0.836).
8. I can turn to my supervisor for help when I need it (0.861).
9. My supervisor tries to solve the problems I face as much as possible (0.900).
10. My supervisor has expertise that can improve my work (0.767).

Social Loafing Behavior (Cronbach’s alpha = 0.851) [7].

1. I defer responsibilities I should assume to other people (0.758).
2. I make less effort on the job when other people are around to do the work (0.691).
3. I do not do my share of the work (0.670).
4. I make less effort than other members of my work group (0.751).
5. I avoid performing housekeeping tasks as much as possible (0.588).
6. I take it easy if other people are around to do the work (0.690).
7. I defer customer service activities to other people if they are present (0.591).

Perceived Organizational Support (Cronbach’s alpha = 0.931) [18].

1. My organization cares about my opinions (0.682).
2. My organization really cares about my well-being (0.655).
3. My organization strongly considers my goals and values (0.686).
4. Help is available from my organization when I have a problem (0.676).
5. My organization shows very little concern for me.(R) (0.749).
6. My organization values my contributions to the organization (0.766).
7. My organization is willing to help me if I need a special favor (0.737).

Perceived Organizational Politics (Cronbach’s alpha = 0.896) [62].

1. When it comes to pay raises and promotion decisions, policies are irrelevant (0.708).
2. Agreeing with powerful others is the best alternative in this organization (0.632).
3. Promotions around here are not valued much, because how they are determined is so political (0.596).
4. I have seen changes made here that only serve the purposes of a few individuals, not the whole work unit or department (0.558).
5. Sometimes it is easier to remain quiet than to fight the system (0.718).
6. Favoritism, rather than merit, determines who gets good raises and promotions around here (0.726).
7. Telling others what they want to hear is sometimes better than telling the truth (0.760).
8. It is safer to think what you are told than to make up your own mind (0.723).
9. Inconsistent with organizational policies, promotions in this organization generally do not go to top performers (0.754).
10. Rewards such as pay raises and promotions do not go to those who work hard (0.574).

Note. Numbers in parentheses are standardized factor loading values.
